# Two biochemically distinct lipophosphoglycans from *Leishmania braziliensis* and *Leishmania infantum* trigger different innate immune responses in murine macrophages

**DOI:** 10.1186/1756-3305-6-54

**Published:** 2013-03-07

**Authors:** Izabela Coimbra Ibraim, Rafael Ramiro de Assis, Natália Lima Pessoa, Marco Antônio Campos, Maria Norma Melo, Salvatore Joseph Turco, Rodrigo Pedro Soares

**Affiliations:** 1 Centro de Pesquisas René Rachou, Fundação Oswaldo Cruz - FIOCRUZ, Av. Augusto de Lima, 1715, Belo Horizonte, 30190-002, Brazil; 2 Departamento de Parasitologia, Universidade Federal de Minas Gerais, Belo Horizonte, MG, Brazil; 3 Department of Biochemistry, University of Kentucky Medical Center, Lexington, KY, USA

**Keywords:** *Leishmania infantum*, *Leishmania braziliensis*, Lipophosphoglycan (LPG), Macrophage modulation

## Abstract

**Background:**

The dominant, cell surface lipophosphoglycan (LPG) of *Leishmania* is a multifunctional molecule involved in the interaction with vertebrate and invertebrate hosts. Although the role of LPG on infection has been extensively studied, it is not known if LPG interspecies variations contribute to the different immunopathologies of leishmaniases. To investigate the issue of interspecies polymorphisms, two *Leishmania* species from the New World that express structural variations of side chains of LPG repeat units were examined. In this context, the procyclic form of *L. braziliensis* LPG (strain M2903), is devoid of side chains, while the *L. infantum* LPG (strain BH46) has up to three glucoses residues in the repeat units.

**Methods:**

Mice peritoneal macrophages from Balb/c, C57BL/6 and knock-out (TLR2 ^−/−^, TLR4 ^−/−^) were primed with IFN-γ and stimulated with purified LPG from both species. Nitric oxide and cytokine production, MAPKs (ERK, p38 and JNK) and NF-kB activation were evaluated.

**Results:**

Macrophages stimulated with *L. braziliensis* LPG, had a higher TNF-α, IL-1β, IL-6 and NO production than those stimulated with that of *L. infantum*. Furthermore, the LPGs from the two species resulted in differential kinetics of signaling via MAPK activation. *L. infantum* LPG exhibited a gradual activation profile, whereas *L. braziliensis* LPG showed a sharp but transient activation. *L. braziliensis* LPG was able to activate NF-kB.

**Conclusion:**

These data suggest that two biochemically distinct LPGs were able to differentially modulate macrophage functions.

## Background

Leishmaniases are a spectrum of diseases widely distributed in the Americas [1]. In Brazil, *Leishmania braziliensis* and *Leishmania infantum* are the causative agents of cutaneous (CL) and visceral leishmaniasis (VL), respectively [2]. At the early steps of infection in the vertebrate host, the parasite must survive the production of inflammatory mediators such as reactive oxygen intermediates (ROI), reactive nitrogen intermediates (RNI), and cytokines [3,4].

In trypanosomatids, many GPI-anchored molecules are known to be closely associated with cell signaling, acting as agonists and second messengers in response to cytokines and other stimuli [5-9]. In *Leishmania,* lipophosphoglycan (LPG) has been extensively studied and is known to be a multifunctional virulence factor with functions that include: attachment to the sand fly vector midgut [10], attachment and entry into macrophages [11], induction of neutrophil extracellular traps (NETs) [12], inhibition of protein kinase C (PKC) [13,14], retardation of phagosome maturation [15], disruption of NADPH oxidase assembly at the phagosome membrane [16] modulation of NO production [17] and induction of protein kinase R and heme oxigenase-1 [18,19]. Although the lipid anchor is conserved, previous studies have shown that changes in the carbohydrate structure of procyclic LPG and GIPLs can account for variations in macrophage modulation [20-22].

**Figure 1:**
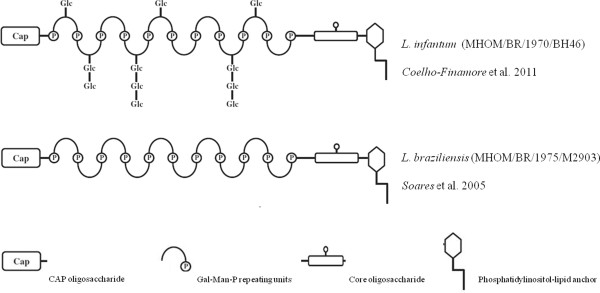
**Biochemical structures of LPG from *L. braziliensis *and *L. infantum.*** Although *Leishmania* LPG shows a well conserved lipid anchor and oligosaccharide core structure, a wide range of polymorphisms of the composition of the sugar branch on the repeat units is well known. For *L. braziliensis* procyclic promastigote LPG, there is no substitution on the repetitive units while in *L. infantum* LPG there are different sugar branch substitutions of glucose oligosacharides that vary from one to three glucoses.

Structurally, LPG has four distinct domains: (i) a well conserved GPI anchor composed of 1-*O*-alkyl-2-*lyso*-phosphatydylinositol (PI); (ii) a core composed of Gal(α1-6)Gal(α1-3)Gal_*f*_(β1-3)[Glc(α1)PO_4_]Man(α1-3)Man(α1-4)-GlcN(α-1) heptasaccharide; (iii) a portion of disaccharide repeats of the Gal(β1-4)Man(α1)PO_4_ units and (iv) a terminal neutral oligosaccharide (“cap”) [11]. In *L. infantum*, the repeat units of LPG display the addition of βGlc to the C3 carbon in the Gal residues [23]. These side chain substitutions can vary between different strains of *L. infantum* which can have three different sets of side chains: Type I which has no side chain substitutions; Type II which has only one βGlc addition to the Gal residue of the repeat units and Type III which contains two or three βGlc residue substitutions on the Gal residue [21] (Figure 1). Interestingly, in the metacyclic promastigote form of LPG in this species there are no sugar substitutions in the repeat unit backbone [23]. Elucidation of the *L. braziliensis* LPG structure showed that procyclic promastigote forms are devoid of side-chains, whereas metacyclic LPG displays up to two βGlc residues linked to the Gal residue of the repeat unit backbone [24]. It is not known to what extent interspecies variations in New World species of *Leishmania* can differentially activate the host’s immune system. Recently, the structural composition of *L. braziliensis* and *L. infantum* GIPLs revealed that the former are galactose rich and its structure is suggestive for the predominance of type II GIPLs having a Manα1-3 substitution to the proximal mannose and are mainly galactose terminating structures. On the other hand, *L. infantum* GIPLs are mainly of type I and/or Hybrid GIPLs possessing Manα1-6 and Manα1-3[Manα6] substitutions to the proximal mannose, respectively [22,25]. Those variations were implicated in a higher inhibitory activity in NO and cytokine production by *L. braziliensis* GIPLs compared to *L. infantum*. However, the role of LPGs from those species in this process has not been investigated.

This work is part of a wider study on the glycobiology of New World species of *Leishmania*. In previous studies, we reported on the LPG and GIPLs biochemical structures of *L. braziliensis* and *L. infantum*[22-24] and showed that the differences in GIPLs structures were relevant in the parasite biology [22]. In this study, we expanded those findings and show that the LPG of these two New World *Leishmania* species also differentially modulated the activation of mouse peritoneal macrophages.

## Methods

### Ethics statement

All animals were handled in strict accordance with animal practice as defined by the Internal Ethics Committee in Animal Experimentation (CEUA) of Fundação Oswaldo Cruz (FIOCRUZ), Belo Horizonte (BH), Minas Gerais (MG), Brazil (Protocol P-0297-06). Knock-out mice handling protocol was approved by the National Commission of Biosafety (CTNBio) (protocol #01200.006193/2001-16).

### Parasites

World Health Organization Reference strains of *L. braziliensis* (MHOM/BR/1975/M2903) and *L. infantum* (MHOM/BR/1970/BH46) were used. Promastigotes were cultured in M199 medium supplemented with 10% heat-inactivated fetal bovine serum (FBS), penicillin 100 units/ml, streptomycin 50 μg/ml, 12.5 mM glutamine, 0.1 M adenine, 0.0005% hemin, and 40 mM Hepes, pH 7.4 at 26°C [23] until late log phase.

### Extraction and purification of LPG

For optimal LPG extraction, late log phase cells were harvested and washed twice with PBS prior to extraction of LPG. The LPG extraction was performed as described elsewhere with solvent E (H_2_O/ethanol/diethylether/pyridine/NH_4_OH; 15:15:5:1:0.017) after a sequential organic solvent extraction [26]. For purification, the solvent E extract was dried under N_2_ evaporation, resuspendend in 2 ml of 0.1 N acetic acid/0.1 M NaCl, and applied onto a column with 2 ml of phenyl-Sepharose, equilibrated in the same buffer. The column was washed with 6 ml of 0.1 N acetic acid/0.1 M NaCl, then 1 ml of 0.1 N acetic acid and finally 1 ml of endotoxin free water. The LPG was eluted with 4 ml of solvent E then dried under N_2_ evaporation. LPG concentration was determined as described elsewhere [27]. Prior to use on *in vitro* macrophage cultures, LPG was diluted in fresh RPMI. All solutions were prepared in sterile, LPS-free distilled water (Sanobiol, Campinas, Brazil).

### Purification of murine peritoneal macrophages and cell culture

Thioglycollate-elicited peritoneal macrophages were extracted from BALB/c, C57BL/6 and C57BL/6 (TLR2^−/−^ and TLR4^−/−^ knockouts) by peritoneal washing with ice cold serum-free RPMI and enriched by plastic adherence for 1 h at 37°C/5% CO_2_. Cells (3 × 10^5^ cells/well) were washed with fresh RPMI then cultured in RPMI, 2 mM glutamine, 50 U/ml of penicillin and 50 μg/mL streptomycin supplemented with 10% FBS in 96-well culture plates (37°C/5% CO_2_). Cells were primed with gamma interferon (IFN-γ) (3 IU/mL) [28] for 18 h prior to incubation with LPG or live stationary *Leishmania* parasites (10:1), LPG (10 μg/mL) or lipopolysaccharide (LPS) (100 ng/mL).

### Chinese Hamster Ovary (CHO) cell lines

The CHO reporter cell lines (TLR2-TLR4-, which do not express TLR2 nor TLR4; TLR2+, expressing TLR2; TLR4+, expressing TLR4 [29] were maintained as adherent monolayers in Ham’s F-12/DMEM supplemented with 5% FBS, at 37°C, 5% CO_2_, and antibiotics. All of the cell lines are derived from clone 3E10, that has been stably transfected with a reporter construct containing the structural gene for CD25 under the control of the human E-selectin promoter. This promoter contains a NF-kB binding site; CD25 surface expression is completely dependent upon NF-kB translocation to the cell nucleus [30].

### Cytokine and nitrite measurements

For CBA multiplex cytokine detection, cells were plated, primed as described above and incubated for 18 h (37°C, 5% CO_2_). LPG (10 μg/mL) and live stationary promastigotes (10:1 ratio) were added and incubated for 48 h, LPS (100 ng/mL) was added as a positive control. For negative controls fresh medium was added. Supernatants were collected and cytokines (IL1-β, IL-2, IL-4, IL-5, IL-6, IL-10, IL-12p40, IFN-γ and TNF-α) were determined using the BD CBA Mouse Cytokine assay kits according to the manufacturer’s specifications (BD Biosciences, CA, USA). Flow cytometry measurements were performed on a FACS Calibur flow cytometer (Becton Dickinson, Mountain View, CA). Cell-Quest™ software package provided by the manufacturer was used for data acquisition and the FlowJo software 7.6.4 (Tree Star Inc., Ashland, OR, USA) was used for data analysis. A total of 1,800 events were acquired for each preparation [22]. Results are representative of two experiments in duplicate. Nitrite concentrations were determined by Griess reaction [31].

For the inhibition assay, purified cells were primed for 6 h with 3 IU/ml of IFN-γ prior to stimulation with LPG (10 μg/ml). Cells were incubated for 18 h at 37°C/5% CO_2_, then LPS (100 ng/ml) was added to the medium and incubated for another 24 h at 37°C/5% CO_2_. Supernatants were collected and nitrite concentrations determined by Griess reaction. Results shown are the mean of two experiments in triplicate [22].

### Flow cytometry analysis

In order to evaluate the activation of NFκB by LPG, CHO reporter cells were plated at a density of 1×10^5^ cells/well in 24-well tissue culture dishes. The following day, either molecule or bacteria (*Staphylococcus aureus* [1000 bacteria/well], positive control of TLR2; LPS (200 ng/well), positive control of TLR4; or LPG (0.2 μg or 0.02 μg/well) from *L. braziliensis or from L. infantum* was added as indicated, for 18 h. The cells were harvested with trypsin-EDTA, washed with medium and with PBS. Subsequently, the cells were counted and 1×10^5^ were cells stained with PE-labeled anti-CD25 (mouse mAb to human CD25, R-PE conjugate; Caltag Laboratories, Burlingame, CA) 1:200 in PBS, on ice, in the dark, for 30 min. After labeling, the cells were washed twice with same buffer, resuspended in 1 mM sodium azide in PBS, and examined by flow cytometry (BD Biosciences, San Jose, CA) for the expression of surface CD25 as described [29]. Analyses were performed using CellQuest software (BD Biosciences).

### Preparation of cell lysates and immunoblotting

Thioglycollate-elicited peritoneal macrophages were plated as above on 6 well tissue culture plates (3 × 10^6^/well) for 18 h prior to assay. The cells were washed with warm RPMI and incubated with LPG from both species for different times (5, 15, 30 and 45 min) or with medium (negative control) or LPS (100 ng/ml) as positive control. Cells were then washed with ice-cold PBS and lysed in lysis buffer (20 mM Tris–HCl pH 7.5, 1% Triton ×-100, 1 mM sodium orthovanadate, 1 mM phenylmethylsulfonyl fluoride (PMSF), 50 mM sodium fluoride, 150 mM NaCl, 5 mM ethylenediamine tetraacetic acid (EDTA), 10% glycerol (v/v), 0.5 mM dithiothreitol (DTT) and protease inhibitor cocktail from Sigma®). Cells were harvested with a plastic scraper and centrifuged at 13,000 × *g* (4°C, 10 min). Supernatants were transferred to new tubes and stored at −20°C until used for immunoblotting. Cell lysates were resolved by SDS-PAGE, transferred to a nitrocellulose membrane and blocked (5% milk in TBS-0.1% Tween 20) for 1 h. Primary Abs (anti-dually phosphorylated ERK, -dually phosphorylated p38 and -Total ERK, 1:1,000, total p38 primary antibody was used as a normalizer) were incubated for 16 h at 4°C. Membranes were washed (3 × 10 min) with TBS-0.1% Tween 20 and incubated 1 h with anti-mouse IgG conjugated with peroxidase (1:10,000). The reaction was visualized using luminol [22].

### Statistical analyses

For nitrite and cytokine measurements, the Shapiro–Wilk test was conducted to test the null hypothesis that data were sampled from a Gaussian distribution [32]. The P value (P > 0.05) showed that data did not deviate from Gaussian distribution. For this reason, Student’s “t” test and ANOVA were performed to test equality of population medians among groups and independent samples. Data were analyzed using GraphPad Prism 5.0 software (Graph Prism Inc., San Diego, CA) and P < 0.05 was considered significant.

## Results

### Nitrite and cytokine production

**Figure 2:**
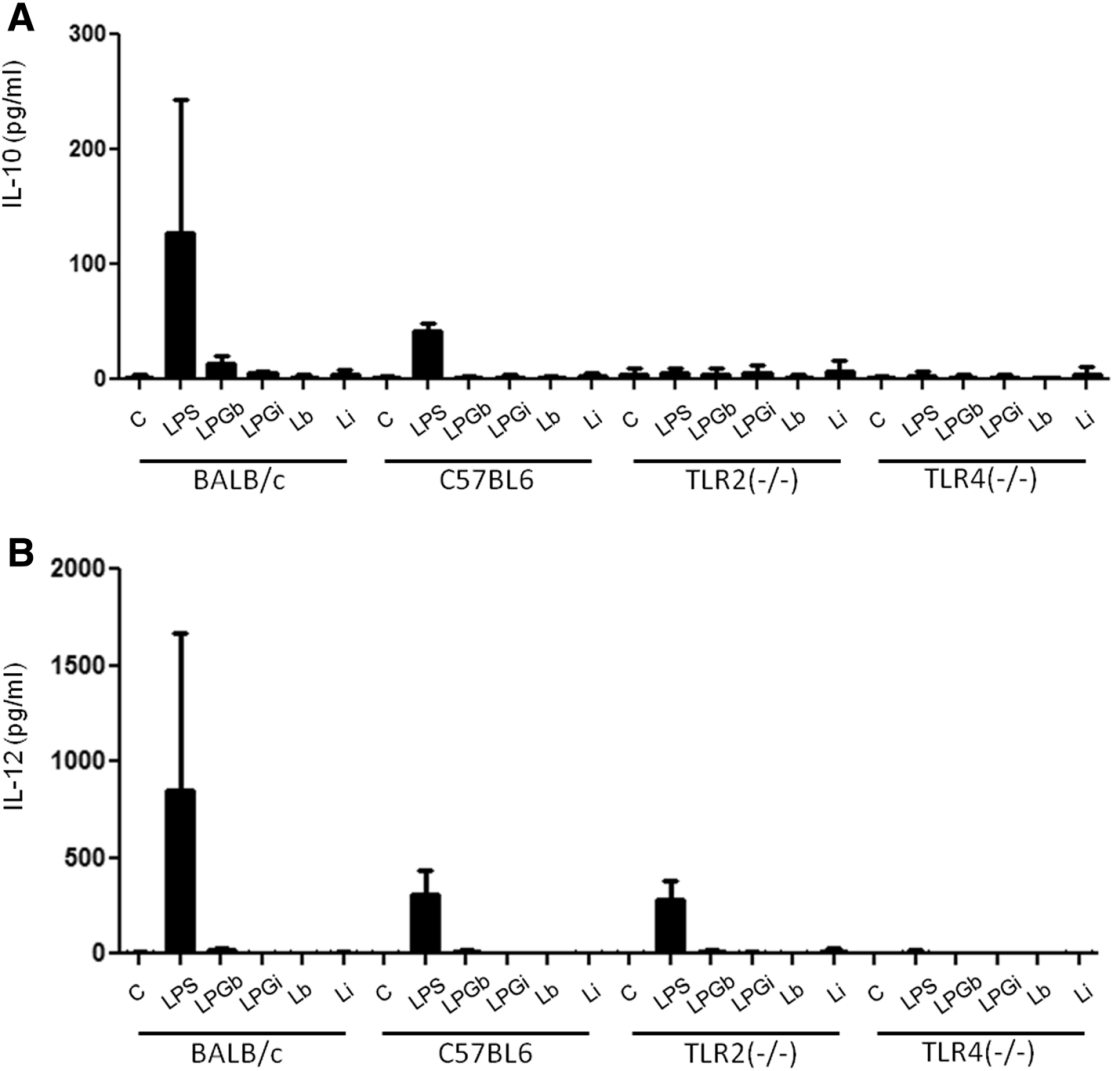
**IL-10 (A) and IL-12 (B) production by IFN-γ primed macrophages stimulated with LPG and live parasites.** C, negative control; LPGb, *L. braziliensis* LPG; LPGi, *L. infantum* LPG; Lb, *L. braziliensis* live promastigotes and Li, *L. infantum* live promastigotes. Cells were pre-incubated with IFN-γ (3 IU/ml) for 18 h then 10 μg/mL of LPG. As a positive control, LPS (100 ng/mL) was added. Fresh medium alone was added to negative control cells. Supernatants were collected 48 hours later, cytokine concentrations determined by flow cytometry. ANOVA test was performed and P < 0.05 was considered significant.

**Figure 3:**
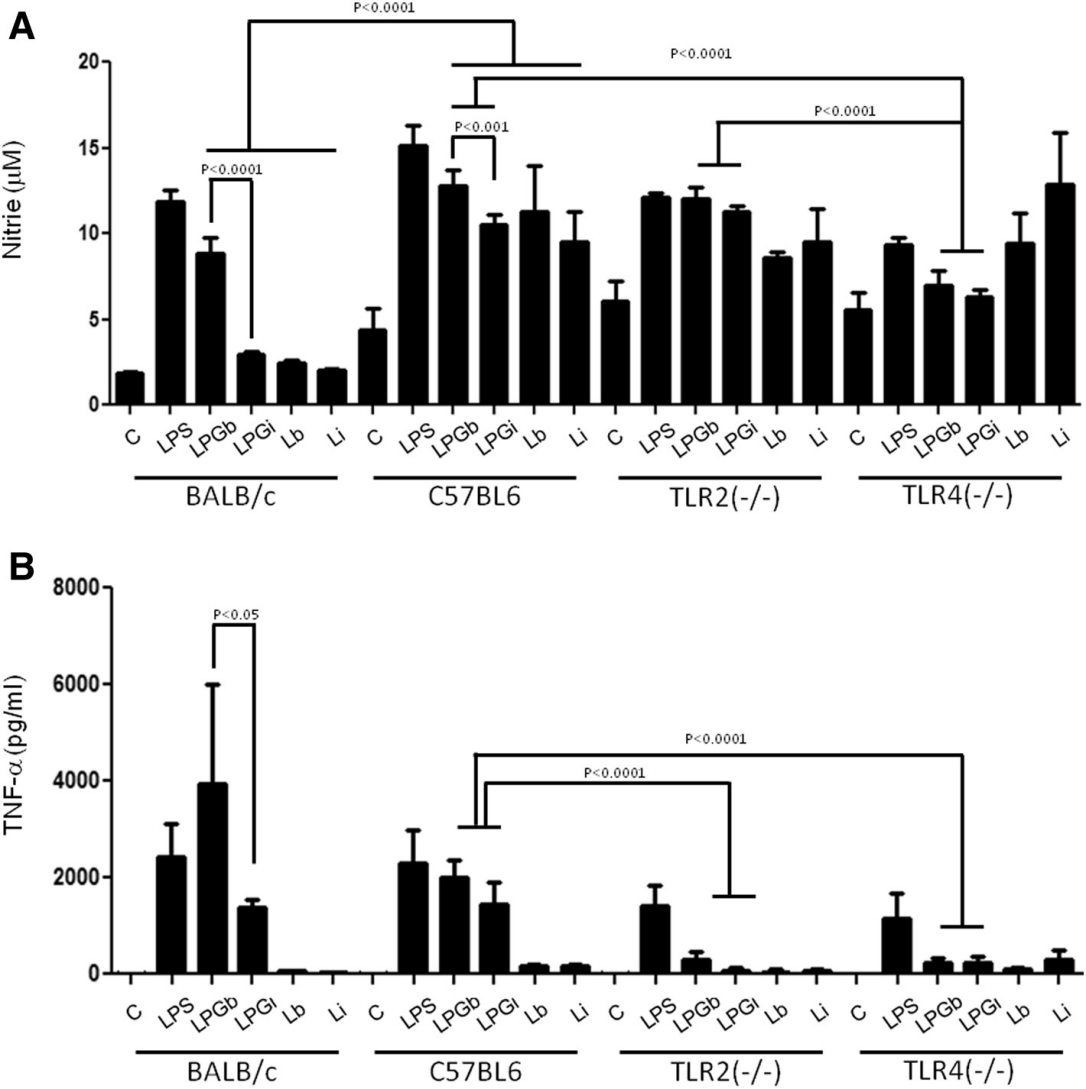
**Nitrite (A) and TNF-α (B) production by IFN-γ primed macrophages stimulated with LPG and live parasites.** C, negative control; LPGb, *L. braziliensis* LPG; LPGi, *L. infantum* LPG; Lb, *L. braziliensis* live promastigotes and Li, *L. infantum* live promastigotes. Cells were pre-incubated with IFN-γ (3 IU/ml) for 18 h then 10 μg/mL of LPG. As a positive control, LPS (100 ng/mL) was added. Fresh medium alone was added to negative control cells. Supernatants were collected 48 hours later. Nitrite concentration was measured by Griess reaction and cytokine concentrations were determined by flow cytometry. ANOVA test was performed and P < 0.05 was considered significant.

**Figure 4:**
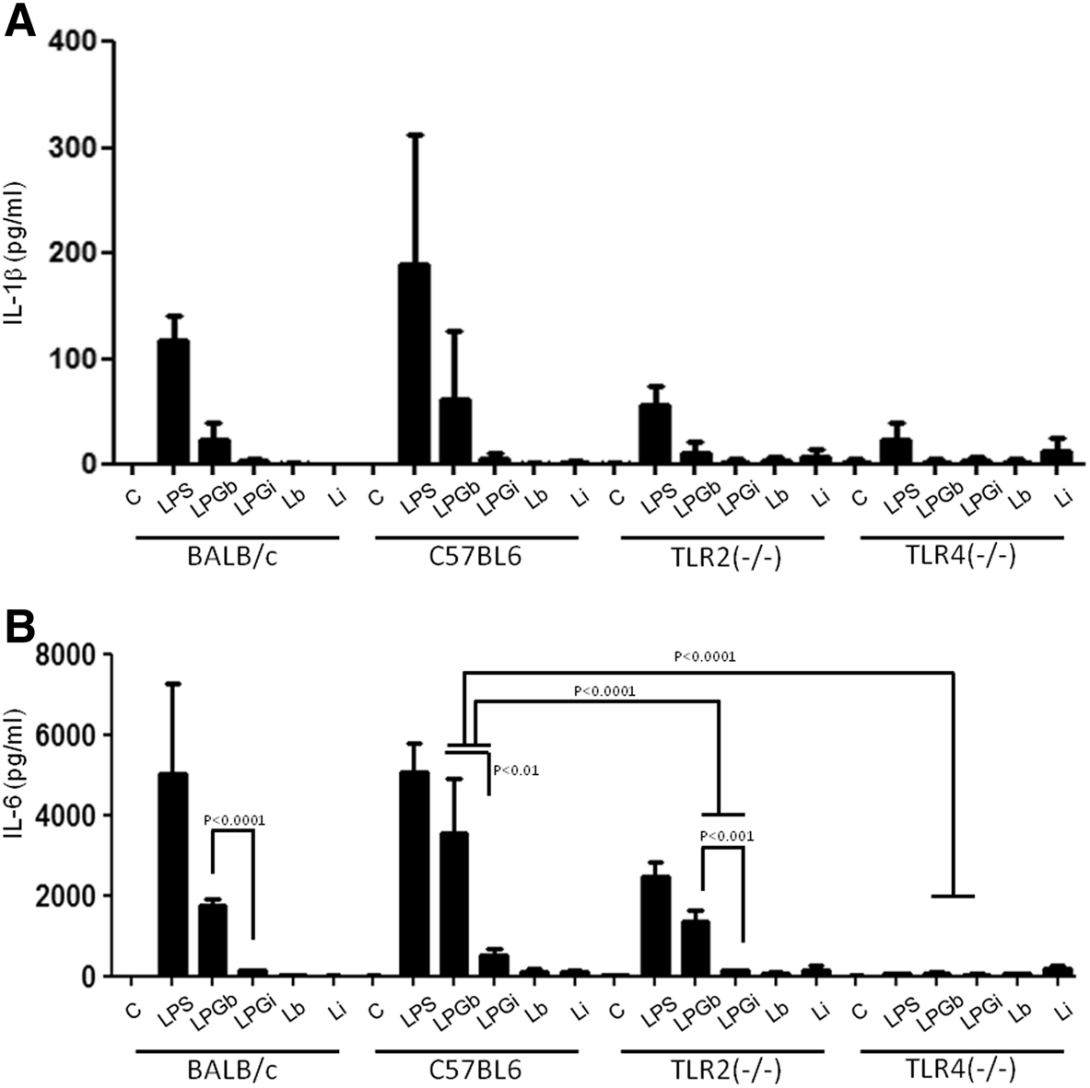
**IL-1β (A) and IL-6 (B) production by IFN-γ primed macrophages stimulated with LPG and live parasites.** C, negative control; LPGb, *L. braziliensis* LPG; LPGi, *L. infantum* LPG; Lb, *L. braziliensis* live promastigotes and Li, *L. infantum* live promastigotes. Cells were pre-incubated with IFN-γ (3 IU/ml) for 18 h then 10 μg/mL of LPG. As a positive control, LPS (100 ng/mL) was added. Fresh medium alone was added to negative control cells. Supernatants were collected 48 hours later, cytokine concentrations were determined by flow cytometry. ANOVA test was performed and P < 0.05 was considered significant.

In order to evaluate the role of TLR2 and TLR4 in this process, IFN-γ primed peritoneal macrophages from BALB/c, C57BL/6, TLR2^−/−^ and TLR4^−/−^ mice were incubated with 10 μg/ml of LPG and live promastigotes (10:1) from *L. braziliensis* and *L. infantum*. Nitrite and cytokine concentrations determined on the supernatants after 48 h (Figures 2, 3 and 4) [22].

A higher NO production was detected on IFN-γ-primed C57BL/6 macrophages stimulated with both LPGs and live promastigotes when compared to macrophages from BALB/c mice (P < 0.001) (Figure 2A). There was a significant decrease in NO production in IFN-γ-primed TLR4^−/−^ macrophages stimulated with LPG in comparison to TLR2^−/−^ and wild type C57BL/6 macrophages (P < 0.01) suggesting the involvement of TLR4 in this activation. No significant difference of NO production was noticed in macrophages from TLR2^−/−^ or TLR4^−/−^ mice stimulated with live promastigotes when compared to wild type C57BL/6 (P < 0.05) (Figure 2A).

In a multiplex flow cytometer approach, the concentrations of several cytokines were determined on the same supernatants used for nitrite measurement. No significant quantities of IL-2, IL-4, IL-5 and IFN-γ were detected (data not shown); on the other hand, a significant production of TNF-α, IL-1β and IL-6 was observed (Figure 3B and 4A and B). As for nitrite production, these cytokines production, in BALB/c and C57BL/6 macrophages, were higher for *L. braziliensis* LPG stimulated cells (P <0.05). In all experiments, live parasites from both species induced cytokine production close to background levels (Figure 3B and 4A and B). As expected, LPG was able to induce these three cytokines in wild type and TLR2^−/−^ mice but not in TLR4^−/−^ mice. A reduction in the production of these cytokines in TLR2^−/−^ macrophages was demonstrated, indicating the importance of this receptor in LPG recognition and signaling. A complete abrogation of the production of these cytokines was observed in TLR4^−/−^ mice suggesting also the participation of TLR4 in LPG recognition and signaling. There was no significant production of IL-10, IL-12p40 (Figure 2) or IFN-γ, IL-2, IL-4 and IL-5 after LPG or live promastigote incubation in any of the mouse strains tested (data not shown).

### Activation of MAPKs

**Figure 5:**
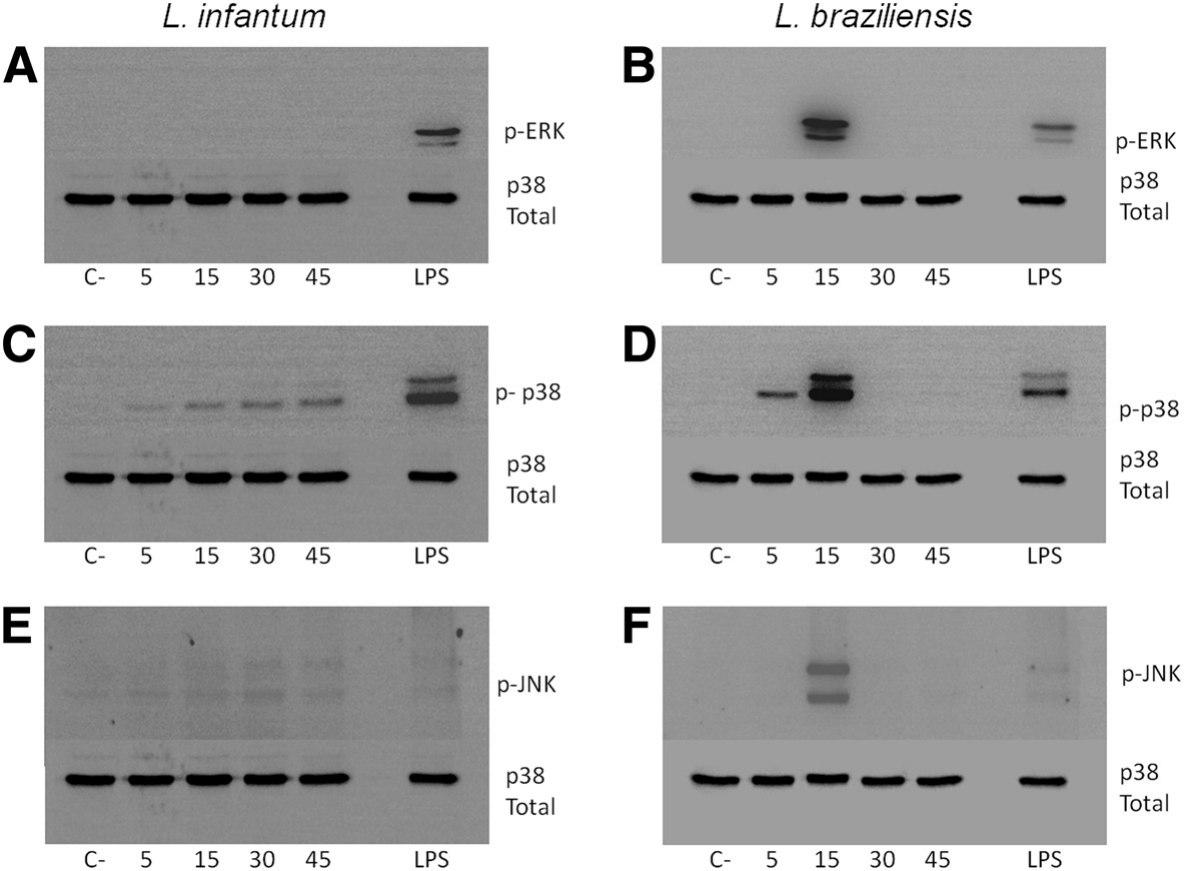
**Activation of MAPKs (ERK, p38 and JNK) by *Leishmania *LPG in BALB/c peritoneal macrophages.** Mouse peritoneal macrophages were stimulated for 45 min with 10 μg/mL of LPG from *L. infantum* (**A**, **C** and **E**) and *L. braziliensis* (**B**, **D** and **F**). Dually phosphorylated MAPKs were detected by western blot. C, negative control; LPGb, *L. braziliensis* LPG and LPGi, *L. infantum* LPG. Total p38 (**A** to **F**) content was used as a normalizing protein.

To better access the signaling events around LPG recognition and macrophage activation, BALB/c macrophages were incubated with *L. braziliensis* and *L. infantum* LPG and MAPK activation was assessed as a function of time. A strong ERK and JNK activation was observed in *L. braziliensis* in LPG stimulated macrophages after 15 min incubation but no activation was detected before or after this time interval. In contrast, no ERK activation by *L. infantum* LPG was found. *L. braziliensis* LPG stimulated a sharp p38 and ERK phosphorylation beginning in 5 minutes and peaking at 15 minutes (Figure 5). In a different activation profile, a progressive increase of p38 and JNK was observed for *L. infantum* LPG stimulated macrophages (Figure 5). Together, these data suggests that the LPG from these two species differentially activates MAPK, which may account for the differences in macrophage nitrite and cytokine production and maybe the reason for differential pathogenesis caused by these two species.

### LPGs from *Leishmania infantum* and from *Leishmania brasiliensis* are agonists of TLR2, inducing *ex vivo* the nuclear translocation of NFkB

**Figure 6:**
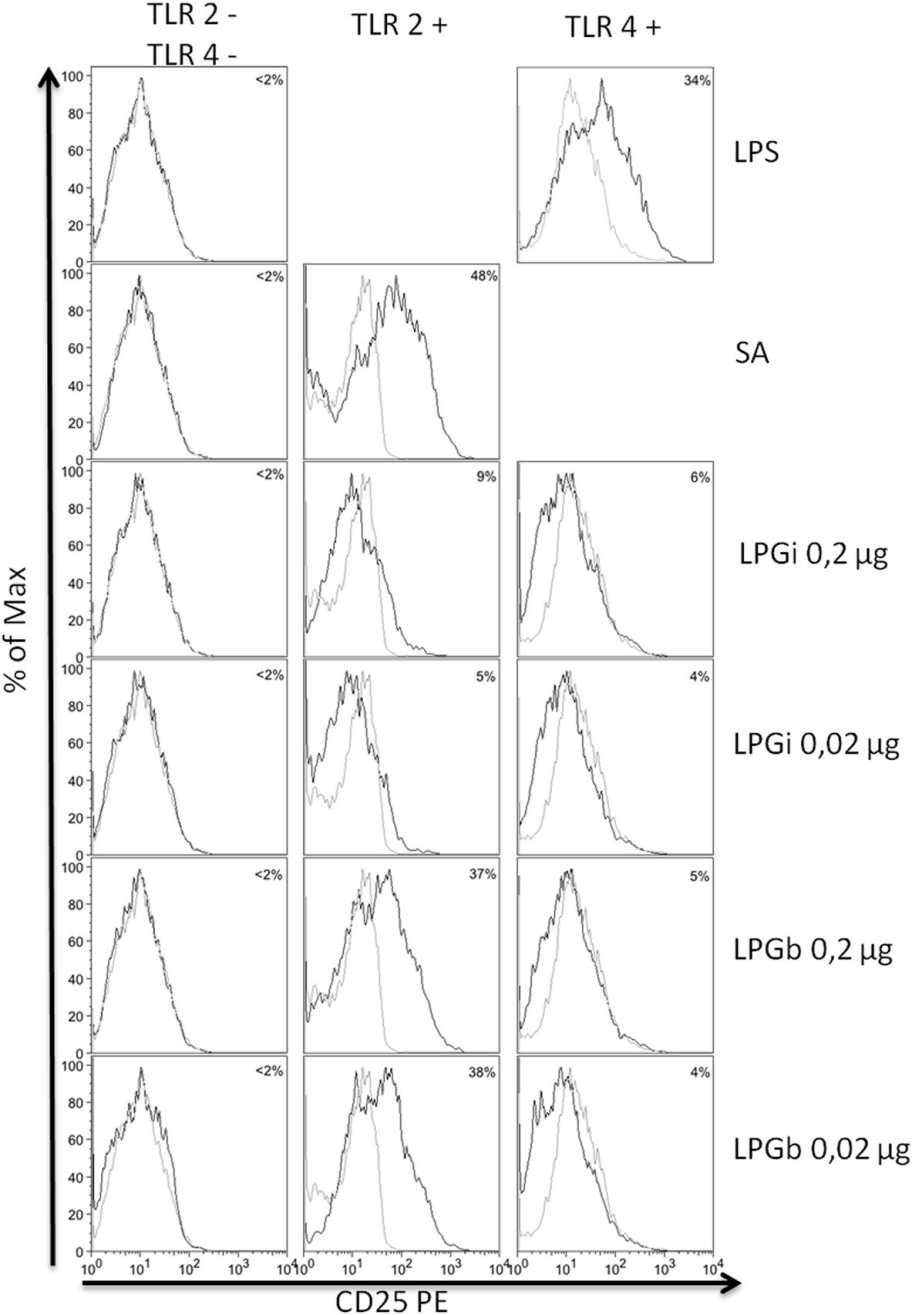
**LPGs purified from *Leishmania infantum *and *Leishmania braziliensis *induce translocation of NFkB through TLRs.** CHO cells expressing TLR2 (TLR2+), TLR4 (TLR4+), or neither (TLR2-/TLR4-) were either untreated (gray line) or exposed (black line) to LPS, *Staphylococcus aureus* (SA), *L. infantum* LPG (LPGi) or *L. braziliensis* LPG (LPGb), as indicated. CD25 expression was measured by flow cytometry 18 h after stimulation. Percentage = percentage of CD25 expression on stimulated cells minus percentage of CD25 expression on non-stimulated cells.

In order to better assess the role of TLR2 and TLR4 in the recognition of LPG, the activation of NFkB was performed. CHO reporter cells were treated with LPG for 18 h and reporter protein CD25 expression evaluated by flow cytometry. As showed in Figure 6, after exposure of CHO cells expressing TLR2 (TLR2+) to 0.2 μg or to 0.02 μg of LPGb, there was a higher production of CD25 protein, resulting from expression of the gene reporter, in comparison with the CHO cells without that exposition, meaning that LPGb is a stark agonist of TLR2, inducing the nuclear translocation of NFkB. After the exposition of TLR4+ cells to LPGb or of TLR4+ or TLR2+ to LPGi, there was a slightly higher production of the protein codified by the gene reporter than the production in the nonstimulated cells, meaning that the LPGb and LPGi are weak agonists of TLR4 and that LPGi is a weak agonist of TLR2.

### Inhibition of nitrite production in BALB/c macrophages by *Leishmania* LPG

**Figure 7:**
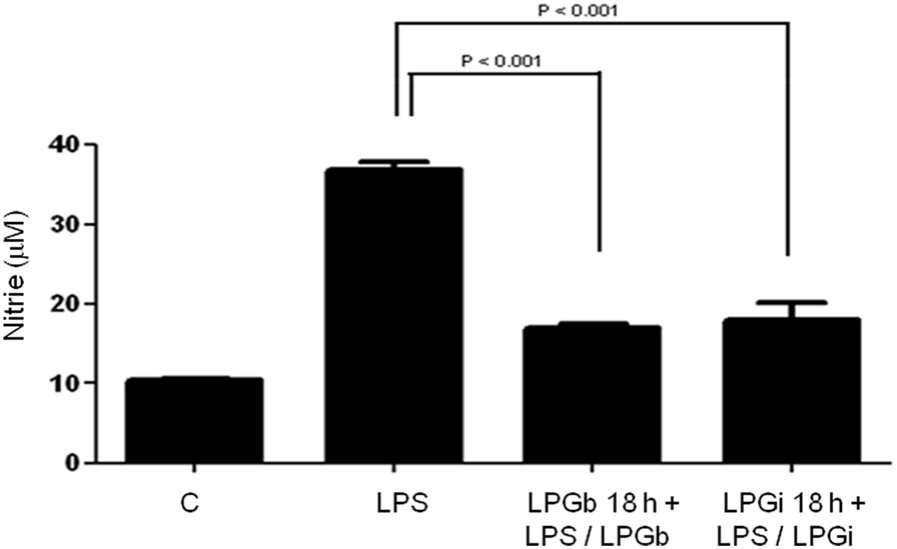
**Modulation of nitrite by *Leishmania *LPG in BALB/c macrophages.** Cells were incubated with LPG (10 μg/ml) from *L. braziliensis* (LPGb) or *L. infantum* (LPGi) for 18 h prior to stimulation with LPS (100 ng/ml) combined with 10 μg/ml of LPG. Cells where then incubated for another 24 h and nitrite content was measured on the supernatants by Griess reaction.

Previously, it was shown that GIPLs from both species were able to inhibit NO production in LPS stimulated mice peritoneal macrophages [22]. To determine whether LPG would also result in inhibitory activity, BALB/c macrophages were incubated in the presence of LPG for 18 h prior to LPS exposure. A strong inhibition of NO production stimulated by LPS was observed after LPG incubation from both species. The inhibition was over 70% (P < 0.01) for LPG from both species (Figure 7).

## Discussion

Leishmaniasis is considered by the World Health Organization as one of the six major infectious diseases in the whole world [33] and affects over 1.5 million people every year worldwide. In Brazil, the majority of visceral and tegumentary cases are due to *L. infantum* and *L. braziliensis*, respectively. Parasite glycoconjugates have long being incriminated in a variety of events during parasite-host interactions modulating important host cell functions [11,22,34,35]. Among these glycoconjugates, LPG is the best studied especially in Old World species of *Leishmania*. In this study, the role of the LPGs, from two epidemiologic important *Leishmania* species in Brazil, in interfering in signaling mechanisms was assessed in murine macrophages.

Since it has been reported that there is no significant difference between procyclic and metacyclic *L. major* LPG NK cell activation [36] and that the conserved GPI anchor is important for LPG activity [32] this work used stationary phase LPG. Although metacyclic promastigotes can be readily prepared from culture by several methods, only a small percentage of parasites, less than 5% [37], differentiate into these forms. For this reason, all experiments described here could not have been done with metacyclic LPG.

One of the most important events in the initial steps of *Leishmania* infection is the production of NO by macrophages. In many models, its production is dependent on a combination of IFN-γ and TNF-α via TLR-dependent mechanisms [38]. Our results indicated that LPG from both species could induce the production of NO in IFN-γ-primed macrophages. Its production was higher in macrophages stimulated with *L. braziliensis* LPG than that of *L. infantum*. C57BL/6 macrophages incubated with both LPGs showed a higher production of NO, IL-1β and IL-6 than BALB/c. On the other hand, TNF-α production after stimulation by *L. braziliensis* LPG was higher in BALB/c macrophages (Figures 2, 3, 4). Similar results were also observed for GIPLs [22], these differences of activation between C57BL/6 and BALB/C mice may be due to the genetic background of mouse strains [37,39]. In the present study, no macrophage activation was observed in WT, TLR2^−/−^ and TLR4^−/−^ murine cells after incubation with live promastigotes.

Previous reports have shown that *in vivo*, pro-inflammatory cytokines such as IL-1β, IL-6 and TNF-α, as well as chemokines, are induced in the initial events of *L. major* and *L. donovani*, causative agents of cutaneous and visceral leishmaniasis infection in the Old World, respectively. Similar results were observed here using *L. braziliensis* and *L. infantum* LPGs since macrophages stimulated with *L. braziliensis* LPG exhibited higher cytokine and NO production compared to that of the visceral form *L. infantum* (Figures 2 and 3). This finding was confirmed after incubation of the LPGs with CHO cells demonstrating that *L. braziliensis* LPG was able to induce NF-kB translocation. These data reflect a similar stimulation pattern between Old World and New World species that causes similar disease outcomes. More importantly, the lack of IL-10, IL-12 production, persistent MAPKs activation and the lack of NF-kB translocation via TLR4 ensure that no traces of endotoxins were present in our preparations.

Because most *Leishmania* glycoconjugates are on the external surface of the cell plasma membrane or secreted, they are able to modulate important functions in cell biology [22]. The interspecies variations observed in *L. infantum* and *L. braziliensis* may be dependent on the action and specificity of glycosyltransferases [23,24]. For example, in *L. donovani* (MONGI strain) critical glycosyltransferases are down regulated in the metacyclic phase [40]. Such intra and interspecies variability is likely to have implications in antigenicity enabling carbohydrates to be important sources of biological diversity [36]. In this work, the differential pattern of macrophage activation might be due to carbohydrate polymorphisms in the LPG of these two species. Our results with the two New World species of *Leishmania* are consistent with the reports from many Old World *Leishmania* species and strains which showed that LPG with its varied structural polymorphisms induced different levels of NO and TNF-α in murine macrophages [20,21,34].

No IFN-gamma, IL-10 or IL-12 production was observed by cells stimulated with LPGs from both New World species. Similar observations can be made in the human visceral leishmaniasis where immune suppression and a mixed Th1/Th2 profile modulate most of the immune response [33]. The lack of IL-12 production by cells stimulated with *Leishmania* GPI-anchored glycoconjugates was also observed elsewhere, where mouse peritoneal macrophages failed to produce IL-12 when co-incubated with *L. braziliensis* or *L. infantum* GIPLs and when also stimulated with IFN-γ or LPS [41]. It is also important to note that the lack of IL-12 production was not due to IL-10 release, since we did not observe any production of this cytokine (Figure 2A). This is similar to that observed when macrophages were treated with synthetic *L. major* LPG [7] and *L. braziliensis* and *L. infantum* GIPLs [22].

In the present work we also evaluated the role of TLRs on the recognition and signaling of LPG. TLR2 was first incriminated as the LPG receptor in macrophages and NK cells [34,42]. Additional *in vivo* experiments demonstrated the importance of TLR3, TLR4 and TLR9 in different *Leishmania* species [43]. By using RNA interference methodologies, it was shown that both TLR2 and TLR3 were implicated in the recognition of *L. donovani* LPG in IFN-γ primed macrophages [44]. *In vivo*, it was shown that TLR4 deficient mice are more susceptible to *Leishmania* infection, failing to efficiently resolve the lesions [45] while TLR2 shows a more regulatory role in *L. braziliensis*-infected dendritic cells [41]. Here, we demonstrated *in vitro* with macrophages and CHO cells that TLR2 and, to a lesser extent, TLR4 were recognized by LPGs from both species clearly suggesting their participation in the LPG signalling. The inability of *L. infantum* LPG to activate NF-kB and ERK could be suggested as an evasion mechanism compared to *L. braziliensis* LPG.

Given that the LPGs were able to induce NO and cytokine production, we investigated whether activation of MAPK signalling was affected. In contrast to GIPLs (which fail to activate MAPKs) [22], LPG from both species activated MAPKs, but with different kinetics. *L. infantum* LPG was not able to activate ERK1/2. In contrast, *L. braziliensis* LPG strongly activated MAPK activity after 15 min. Interestingly, p38 and JNK activation exhibited a gradual and transient profile in *L. infantum* and *L. braziliensis,* respectively (Figure 5). Although a punctual MAPK activation was observed for *L. braziliensis* LPG after 5 min, this may not be a sufficient stimulus for IL-12 production. Activation of p38 appears to be important for controlling *Leishmania* infection since anisomycin reduced parasite survival upon p38 activation [46]. Feng et al. [7] reported that ERK1/2 and p38 are important for NO and TNF-α production by macrophages stimulated with LPS. Consistent with those observations, our data suggests that the LPGs from New World species are also able to differentially activate MAPKs.

The data presented here demonstrated a differential production of NO, cytokines and MAPK activation profile by *L. braziliensis* and *L. infantum* LPG stimulated macrophages. The LPG from these species have a limited proinflammatory potential since they fail to activate important proinflammatory cytokines such as IL-12 and only activate low amounts of IL-1β while inducing activation of early inflammatory cytokines such as TNF-α and IL-6. Additionally, as seen in Figure 7, preincubation with LPG prior to stimulation with LPS reduced the nitrite production to basal levels, indicating that the dynamics of infection must be well regulated and consistent with the long-known proposal that LPG acts as a multifunctional virulence factor for *Leishmania*.

## Conclusion

Together with the complex interface of interaction between parasite and host, glycoconjugate interspecies polymorphisms, not only in the LPG, but also in GIPLs, gp63 and other GPI-anchored molecules, are important for differential regulation of initial events of the immune response as well as establishment of infection. Our results with New World species are consistent with this issue of the importance of LPG polymorphisms; structural variations in LPG resulted in differential activation of macrophages (NO, cytokines and MAPKs). Those polymorphisms could result in different clinical outcomes, such as those shown by *L. infantum* and *L. braziliensis*, causative agents of visceral and tegumentary forms, respectively [47].

## Competing interests

The authors declare that they have no competing interests.

## Authors’ contributions

Conceived and designed the experiments: ICI, RRA, RPS, MNM, MAC, SJT; performed the experiments: ICI, RRA, NLM, MAC; analyzed the data: ICI, RRA, MAC, RPPS; helped to draft the manuscript: ICI, RRA, MNM, MAC, NLM, SJT, RPS. All authors read and approved the final manuscript.
